# Feasibility and clinical value of nurse-led point-of-care ultrasound screening for prethrombotic state in postoperative cardiac ICU patients: a cross-sectional study

**DOI:** 10.1186/s13089-025-00459-9

**Published:** 2025-10-14

**Authors:** Jingjing Cheng, Lu Liu, Xuemei Tang, Chunlin Xiang, Yiwei Qin, Yi Zhang, Zhenjie Jiang, Xiaoting Zeng, Ying Wu, Xiaoyu Chen, Fengchun Liu, Xiaoxiao Wu, Yiping Wang

**Affiliations:** 1 Department of Intensive Care Medical Center, Sichuan Provincial People’s Hospital, School of Medicine, University of Electronic Science and Technology of China, Chengdu, China; 2 The First Affiliated Hospital of Chengdu Medical College, Chengdu, China; 3 Department of Respiratory and Critical Care Medicine, Wenjiang District People’s Hospital, Chengdu, China; 4 Department of Intensive Care Unit, Chengdu Fourth People’s Hospital, Chengdu, China

**Keywords:** Nurse-led POCUS, Prethrombotic state, Cardiac surgical procedures, Interprofessional relations, Critical care nursing

## Abstract

**Background:**

Postoperative cardiac ICU patients are at increased risk of developing venous thromboembolism (VTE), yet early identification of prethrombotic state (PTS) remains challenging. Nurse-led point-of-care ultrasound (POCUS) offers a non-invasive, real-time tool for vascular screening that may enhance early risk stratification.

**Objectives:**

To determine the prevalence of PTS in postoperative cardiac ICU patients using a nurse-led POCUS protocol, identify associated clinical risk factors, and evaluate the feasibility and clinical value of integrating this screening model into routine nursing care within an interprofessional framework.

**Methods:**

This cross-sectional study included 123 adult patients following cardiac surgery. ICU nurses trained in POCUS independently performed bedside color Doppler scans to detect the “blizzard” sign in lower limb veins. Demographic, clinical, and laboratory data were collected. Multivariate logistic regression was used to identify independent predictors of PTS. The nurse-led POCUS protocol was implemented collaboratively with doctors, who provided image verification and clinical oversight as needed.

**Results:**

The “blizzard” sign was observed in 61.8% of patients, most commonly in the popliteal and posterior tibial veins. Nine patients developed deep vein thrombosis, all of whom had prior severe, diffuse-type “blizzard” signs. Independent predictors of PTS included prolonged bed rest (OR 1.016, *P* = 0.031), elevated peak platelet count (OR 1.007, *P* = 0.041), and increased postoperative C-reactive protein levels (OR 1.015, *P* < 0.001). The nurse-led POCUS protocol was implemented successfully with doctor oversight and demonstrated high feasibility and diagnostic yield.

**Conclusion:**

Nurse-led POCUS is a feasible, non-invasive approach for early identification of PTS in postoperative cardiac ICU patients. Embedding such screening protocols into nursing workflows may support timely thromboprophylaxis, expand nursing roles in advanced assessment, and enhance interprofessional ICU care delivery.

**Supplementary Information:**

The online version contains supplementary material available at 10.1186/s13089-025-00459-9.

## Introduction

Deep vein thrombosis (DVT) is a common and potentially life-threatening complication following cardiac surgery [1]. Cardiac surgical patients in intensive care unit (ICU) are at high thrombotic risk due to factors such as prolonged operative time, cardiopulmonary bypass (CPB), sedation, mechanical ventilation, and immobility—all of which promote venous stasis and hypercoagulability [2–4]. However, early thrombotic changes are often silent, making timely identification challenging.

Although risk assessment tools like the Caprini and Padua scores are widely used perioperatively, their specificity is limited in cardiac surgical patients, who are typically classified as uniformly high-risk [5]. This reduces their utility for individualized prevention in this vulnerable population.

Point-of-care ultrasound (POCUS) has become an increasingly valuable bedside tool for thrombotic risk screening in critical care. Nurse-led POCUS, in particular, is safe, efficient, and well-suited for early vascular assessment without overburdening centralized imaging services [6, 7]. When embedded within an interprofessional care model, it enables high-risk patients to be identified timely and facilitates shared decision-making for early, patient-specific interventions.

The prethrombotic state (PTS), marked by slow venous flow and red blood cell aggregation, represents a subclinical stage before overt DVT [8]. The “blizzard” sign—an echogenic swirling pattern seen on Doppler ultrasound—may serve as an early indicator of PTS. However, little is known about its prevalence and predictive value in postoperative cardiac ICU patients, particularly when assessed by nurses at the bedside.

This study aims to evaluate the prevalence of PTS in postoperative cardiac ICU patients using nurse-led POCUS protocol, supported by doctor oversight, and to identify clinical predictors that may guide early, individualized thrombosis prevention in the context of interprofessional care.

## Methods

### Study design and setting

This cross-sectional study was conducted from September 2023 to March 2024 in an ICU of a tertiary hospital in southwest China. The study aimed to assess the prevalence of PTS in postoperative cardiac patients and identify associated risk factors, using nurse-led POCUS within an interprofessional care model.

### Participants

A total of 123 adult patients who underwent cardiac surgery and remained in the ICU for at least 24 h were consecutively enrolled. Inclusion criteria encompassed patients, 18 years or older, admitted to the ICU postoperatively. Exclusion criteria included: (1) Patients not receiving postoperative anticoagulation therapy. (2) Allergy to ultrasound coupling agents. (3) Death within 24 h post-surgery. (4) Incomplete clinical data. (5) Pregnant or postpartum women.

The sample size was determined by using the rule of thumb for cross-sectional studies, which recommends a minimum of 5–10 participants per variable in multivariate analysis. With 19 variables considered, the estimated sample size ranged from 95 to 190 participants. Accounting for a potential 20% dropout rate, the adjusted required sample size was between 114 and 228. Ultimately, 123 participants were included, meeting the estimated requirement.

### Nurse training and ultrasound protocol

Prior to the study, ICU nurses completed the standardized vascular ultrasound training program organized by the Chinese Critical Care Ultrasound Study Group (CCUSG). The program included 2 days of theoretical instruction on ultrasound physics, vascular anatomy, and image interpretation, followed by a supervised practice phase. Within 3 months, each nurse was required to independently perform and interpret 10 lower limb Doppler scans for PTS assessment under teacher confirmation before certification. After certification, nurses routinely applied vascular ultrasound in ICU practice, gaining additional experience, and all examinations in this study were independently performed by these certified nurses.

**Fig. 1:**
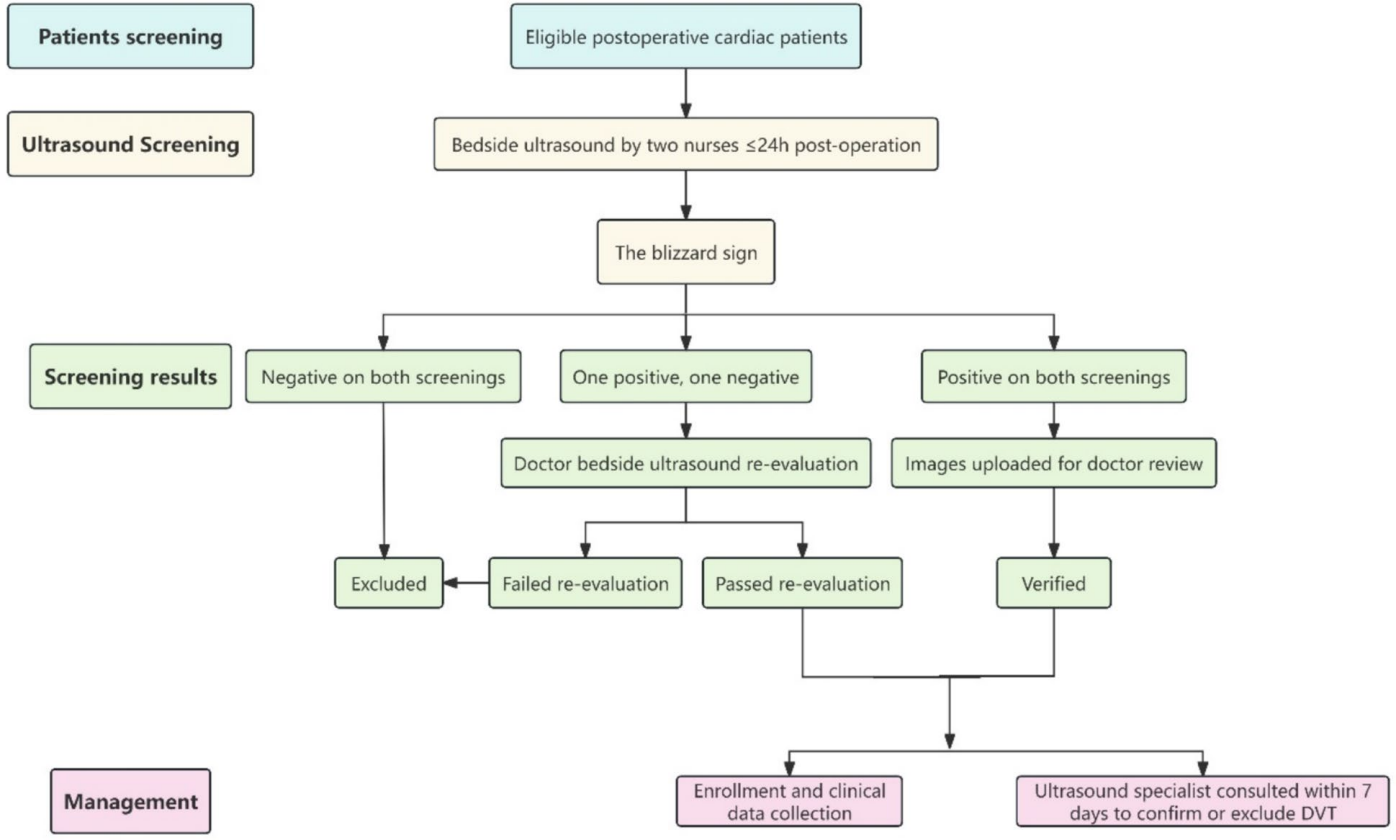
Operational flowchart of the study protocol

During this study, the presence of the “blizzard” sign was first assessed independently by two nurses within 24 h after cardiac surgery. If both nurses identified the sign, the ultrasound images were uploaded to the cloud for doctor review. In cases of disagreement between the two nurses, a doctor performed a bedside re-examination to make the final determination regarding the presence of the “blizzard” sign. Once a patient was identified as being in PTS, a senior ultrasound doctor was contacted, and an ultrasound examination for DVT was completed within 7 days to confirm the presence or absence of thrombosis. The operational flowchart is shown in Fig. 1.

### Color Doppler ultrasound examination method

Certified ICU nurses conducted bedside color Doppler ultrasound examinations using a porTable device (M9, Mindray) equipped with a 12–14 MHz linear-array transducer. Patients were positioned supine with knees slightly flexed. After applying coupling gel, the gain was adjusted to enhance sensitivity. Bilateral assessments included the femoral, popliteal, posterior tibial, peroneal, and intermuscular veins.

**Fig. 2:**
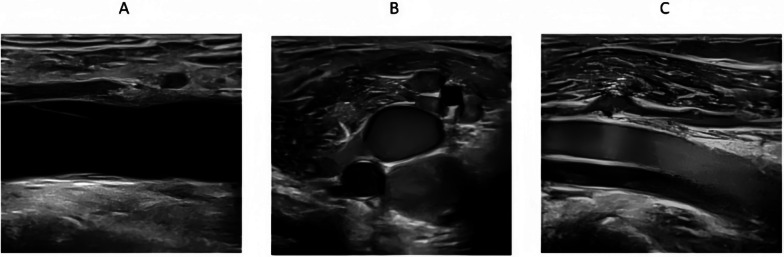
Representative ultrasound images of vascular sections with and without the “blizzard” sign. **A** Long-axis view of a lower limb vein without the “blizzard” sign. **B** Short-axis view showing hyperechoic swirling echoes consistent with the “blizzard” sign. **C** Long-axis view with visible “blizzard” sign indicating red blood cell aggregation due to venous stasis

During the examination, gentle probe pressure was applied to visualize the venous lumen clearly. The presence of flocculent or foggy hyperechoic signals with slow movement within the vein lumen was identified as the “blizzard” sign (Fig. 2, Supplementary material 1 & 2), indicative of red blood cell aggregation due to venous stasis. Despite this appearance, vein compressibility remained intact, and color Doppler imaging showed no flow defects.

The intensity of hyperechoic signals was categorized as mild, moderate, or severe based on the quantity observed. Additionally, the distribution patterns were classified into marginal, central, or diffuse types [9].

### Data collection and analysis

Demographic and clinical data, including age, sex, duration of bed rest, peak platelet count, and initial postoperative C-reactive protein levels, were collected from electronic medical records. Multivariate logistic regression analysis was performed to identify independent risk factors associated with PTS. Statistical significance was set at *P* < 0.05.

### Statistical analysis

Data were analyzed using SPSS Statistics version 24.0 (IBM Corp., Armonk, NY, USA). Continuous variables were assessed for normality using the Shapiro–Wilk test. Normally distributed data are presented as mean ± standard deviation and compared using the independent samples t-test. Non-normally distributed data are expressed as median and interquartile range and analyzed using the Wilcoxon rank-sum test. Categorical variables are reported as frequencies and percentages and compared using the chi-square test. Multivariate logistic regression analysis was conducted to identify independent risk factors associated with PTS. A two-tailed P-value of less than 0.05 was considered statistically significant.

### Ethical consideration

The study was conducted in accordance with the Declaration of Helsinki and adhered to Chinese regulations on clinical trial research. Ethical approval was obtained from the institutional review board of the participating hospital. Written informed consent was obtained from all participants or their legally authorized representatives prior to enrollment.

## Results

### Prevalence and distribution of the “blizzard” sign

#### Incidence and location of the “blizzard” sign in postoperative cardiac ICU patients incidence and anatomical distribution

Among the 123 postoperative cardiac ICU patients, the “blizzard” sign was detected in 76 individuals, yielding an incidence rate of 61.8%. This cohort comprised 35 females and 41 males, aged between 18 and 80 years, with a mean age of 56.74 ± 11.19 years. The types of cardiac surgeries performed included 36 valvular surgeries, 17 coronary artery bypass grafting procedures, 15 major vascular surgeries, 4 congenital heart defect repairs, and 4 other cardiac operations.

In total, 246 nurse-led ultrasound screenings were completed for the 123 enrolled patients. Doctor re-examinations were conducted only in cases where discrepancies were noted between the two nurses.

**Fig. 3:**
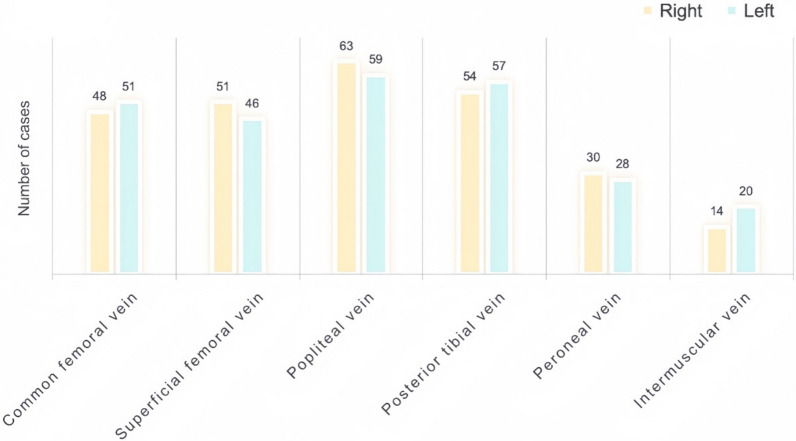
Comparative distribution of the “blizzard” sign in lower extremity veins

A total of 521 “blizzard” sign occurrences were documented—261 on the left side and 260 on the right. The popliteal vein exhibited the highest frequency of this sign, followed by the posterior tibial vein. These findings are illustrated in Fig. 3.

The “blizzard” sign was most frequently observed in the popliteal and posterior tibial veins, with no significant difference in occurrence between the left and right legs.

#### Severity grading

Among the 76 patients exhibiting the “blizzard” sign, severity grading was as follows: 1 case (1.3%) presented with mild intensity alone, 60 cases (78.9%) with severe intensity alone, and 15 cases (19.7%) displayed a combination of mild, moderate, and severe intensities across different venous segments. Notably, mild and moderate “blizzard” signs were predominantly observed in the femoral vein, particularly within the common femoral vein.​

#### Morphological patterns

**Fig. 4:**
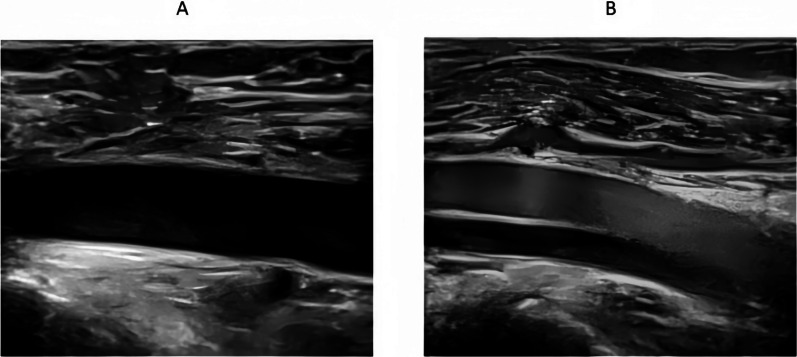
Classification of the “blizzard” sign on Doppler ultrasound. The marginal type “blizzard” sign is characterized by hyperechoic swirling echoes localized along the vessel wall. The diffuse type presents as dense, echogenic signals distributed throughout the venous lumen

Among the 76 patients who exhibited the “blizzard” sign, 12 (15.8%) showed a marginal-type pattern, while 75 (98.7%) presented with the diffuse type. One patient displayed a marginal-type “blizzard” sign confined to the right superficial femoral vein. The remainder exhibited either the diffuse type or a combination of both patterns. Detailed distributions are summarized in Table 1, and representative ultrasound images are shown in Fig. 4 and Supplementary Materials 3 and 4.

**Table 1:** Classification and grading of the “blizzard” sign in lower limb veins

	Simple type (case)	Mixed type (case)	Veins
Marginal type	1	0	Superficial femoral vein
Diffuse type	64	11	All veins

The sign was categorized based on its distribution pattern (marginal, central, diffuse) and severity (mild, moderate, severe) as visualized by Doppler ultrasound

#### Dynamic characteristics and valve involvement

**Fig. 5:**
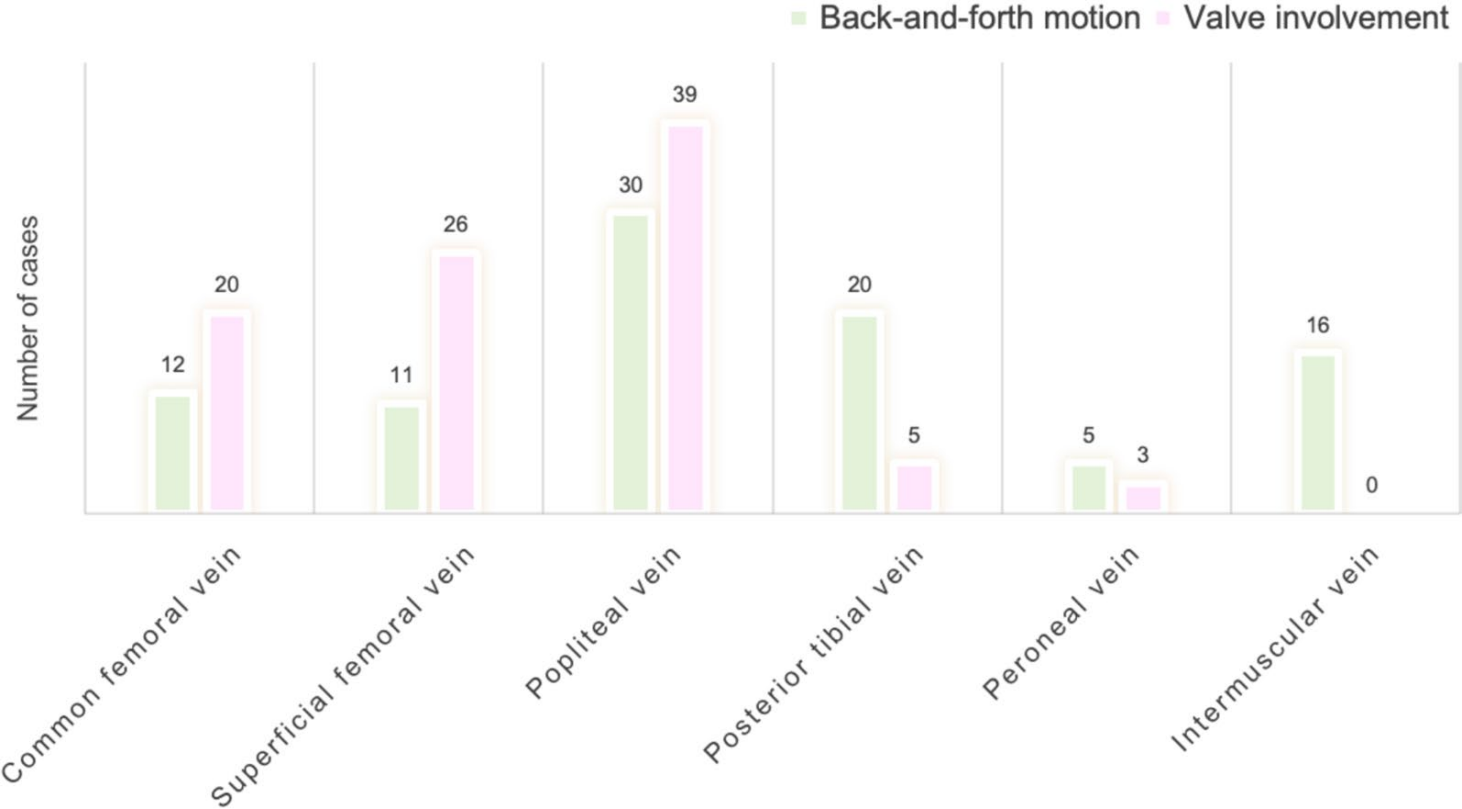
Distribution of the “blizzard” sign by back-and-forth motion and valve involvement in lower extremity veins. The “blizzard” sign was most frequently detected in the popliteal, posterior tibial, and superficial femoral veins. Back-and-forth flow patterns were most prominent in the popliteal and posterior tibial veins, while valve-associated findings were predominantly observed in the popliteal and superficial femoral veins

**Fig. 6:**
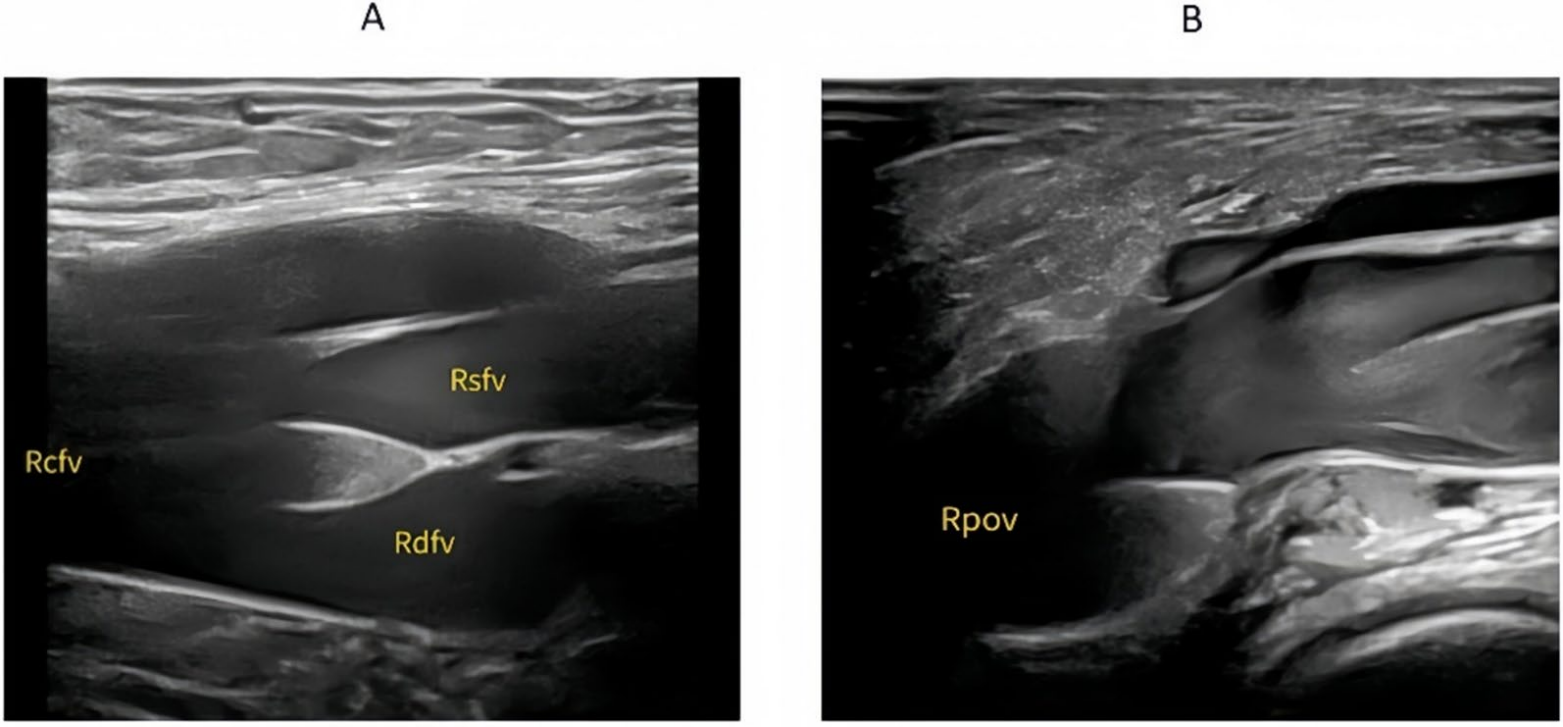
Valve-associated “blizzard” signs in lower extremity veins. Representative Doppler ultrasound images showing “blizzard” signs localized in the femoral and popliteal veins. Rcfv: right common femoral vein; Rsfv: right superficial femoral vein; Rdfv: right deep femoral vein; Rpov: right popliteal vein

The back-and-forth motion of the “blizzard” sign was predominantly observed in the popliteal vein, followed by the posterior tibial vein. Valve-associated “blizzard” signs were most frequently detected in the popliteal vein, followed by the superficial femoral veins, particularly at venous confluence points. These findings are illustrated in Figs. 5 and 6, as well as in Supplementary Material 5.

### Statistical analyses

#### Univariate analysis of associated factors

Univariate analysis identified several factors significantly associated with the presence of the “blizzard” sign (*P* < 0.05): hypertension, duration of bed rest, dialysis status, interleukin-6 levels, postoperative D-dimer levels on day 1 and peak levels within 7 days, peak platelet count within 7 days, and C-reactive protein levels on day 1 and peak levels within 7 days. Conversely, no significant associations were observed for sex, age, diagnosis, diabetes, body mass index (BMI), duration of mechanical ventilation, Caprini score, timing and dosage of anticoagulant therapy, intra-aortic balloon pump (IABP) support, hematocrit, initial postoperative platelet count, white blood cell count, or procalcitonin levels (*P* > 0.05). Detailed results are provided in Additional file: Table 2.

**Table 2:** Univariate analysis of the “blizzard” sign in postoperative cardiac ICU patients

Category	“Blizzard” sign		x^2^/t/z value	*P* value
	Negative cases (*n* = 47)	Positive cases (*n* = 76)		
Sex			0.144	0.704
Man	27 (57.45)	41 (53.95)		
Woman	20 (42.55)	35 (46.05)		
Age	55.43 ± 10.30	56.74 ± 11.19	0.651	0.516
Main diagnosis			7.69	0.243
Congenital heart disease	3 (6.38)	4 (5.26)		
Coronary artery disease	8 (17.02)	17 (22.37)		
Valvular heart disease	27 (57.45)	36 (47.37)		
Cardiac tumor	0 (0.00)	2 (2.63)		
Heart and large vessel disease	4 (8.51)	15 (19.74)		
Endocarditis	5 (10.64)	2 (2.63)		
Duration of bed rest (h)*	50 (IQR 54)	111 (IQR 161)	– 3.032	0.002
Duration of mechanical ventilation (h)*	17 (IQR 7)	18 (IQR 73)	– 1.386	0.166
Caprini score	6.84 ± 1.27	6.51 ± 1.20	1.441	0.152
Starting time of anticoagulant use (h)	14.96 ± 8.70	17.09 ± 14.55	0.909	0.365
IABP support			1.347	0.246
NO	42 (89.36)	62 (81.58)		
Yes	5 (10.64)	14 (18.42)		
Dialysis status			6.459	0.011
NO	46 (97.87)	63 (82.89)		
Yes	1 (2.13)	13 (17.11)		
Hypertension			3.903	0.048
Absent	36 (76.60)	45 (59.21)		
Present	11 (23.40)	31 (40.79)		
Diabetes			2.40	0.121
Absent	44 (93.62)	64 (84.21)		
Present	3 (6.38)	12 (15.79)		
BMI	23.56 ± 3.63	24.28 ± 3.92	1.009	0.315
Interleukin-6 level*	129.96 (IQR 239.76)	167.25 (IQR 341.94)	– 2.082	0.037
D– dimer levels*				
Peak values within 7 days postoperatively	1.94 (IQR 4.31)	7.87 (IQR 8.47)	– 2.452	0.014
On the first postoperative day	0.91 (IQR 1.32)	1.75 (IQR 2.25)	– 3.446	0.001
Hematocrit				
Peak values within 7 days postoperatively	38.05 ± 7.29	37.45 ± 4.49	– 0.555	0.58
On the first postoperative day	35.32 ± 6.93	37.02 ± 4.68	1.618	0.108
Platelet count				
Peak values within 7 days postoperatively	190.04 ± 72.62	242.25 ± 87.17	– 3.433	0.001
On the first postoperative day	144.95 ± 60.17	162.80 ± 64.07	1.537	0.127
White blood cell count				
Peak values within 7 days postoperatively	18.80 ± 5.85	19.82 ± 8.27	0.798	0.426
On the first postoperative day	16.39 ± 5.69	17.69 ± 5.88	1.206	0.23
C-reactive protein levels				
Peak values within 7 days postoperatively	157.41 ± 87.42	189.09 ± 64.41	2.307	0.023
On the first postoperative day	89.08 ± 90.44	178.61 ± 75.64	5.88	< 0.001
Procalcitonin levels*				
Peak values within 7 days postoperatively	1.40 (IQR 3.29)	2.08 (IQR 6.12)	– 0.341	0.733
On the first postoperative day	0.58 (IQR 2.26)	1.00 (IQR 3.98)	– 1.429	0.153

Comparison of demographic, clinical, and laboratory characteristics between patients with and without the “blizzard” sign

^*^Indicates skewed data,which is described in the table using the median and interquartile range

#### Multivariate logistic regression

The presence of the “blizzard” sign (0 = absent, 1 = present) was used as the dependent variable in binary logistic regression analysis. Independent variables with statistical significance in univariate analysis (*P* < 0.05)—including hypertension (0 = no, 1 = yes), duration of bed rest, dialysis status (0 = no, 1 = yes), interleukin-6 level, postoperative D-dimer levels on day 1 and peak levels within 7 days, peak platelet count within 7 days, and C-reactive protein (CRP) levels on day 1 and peak levels within 7 days—were entered into the model.

Categorical variables were coded accordingly, and continuous variables were included as raw values. The Hosmer–Lemeshow goodness-of-fit test indicated an adequate model fit (χ^2^ = 3.220, *P* = 0.920), suggesting no significant difference between predicted and observed values.

The regression analysis identified duration of bed rest, peak platelet counts within the first 7 postoperative days, and initial postoperative CRP level as independent predictors of the “blizzard” sign (*P* < 0.05; see Table 3).

**Table 3:** Logistic regression analysis of the “blizzard” sign in postoperative cardiac ICU patients

Variables	*β* value	Standard error	Wald x^2^ value	*P* Value	*OR* value	95%*CI*
Duration of bed rest	0.015	0.007	4.665	0.031	1.016	1.001–1.031
Peak platelet count within the first 7 postoperative days	0.007	0.003	4.177	0.041	1.007	1.001–1.013
Initial postoperative C-reactive protein level	0.015	0.004	12.517	< 0.001	1.015	1.007–1.023

### Confirmed DVT cases

Among the 123 postoperative cardiac ICU patients who received standard pharmacological prophylaxis, 9 (7.3%) developed DVT. This group included 6 males and 3 females. Surgical procedures among these patients comprised 4 major vascular surgeries, 3 coronary artery bypass grafting procedures, and 2 valvular surgeries. All 9 patients experienced prolonged bed rest exceeding 7 days, with one patient bedridden for over 37 days.

**Fig. 7:**
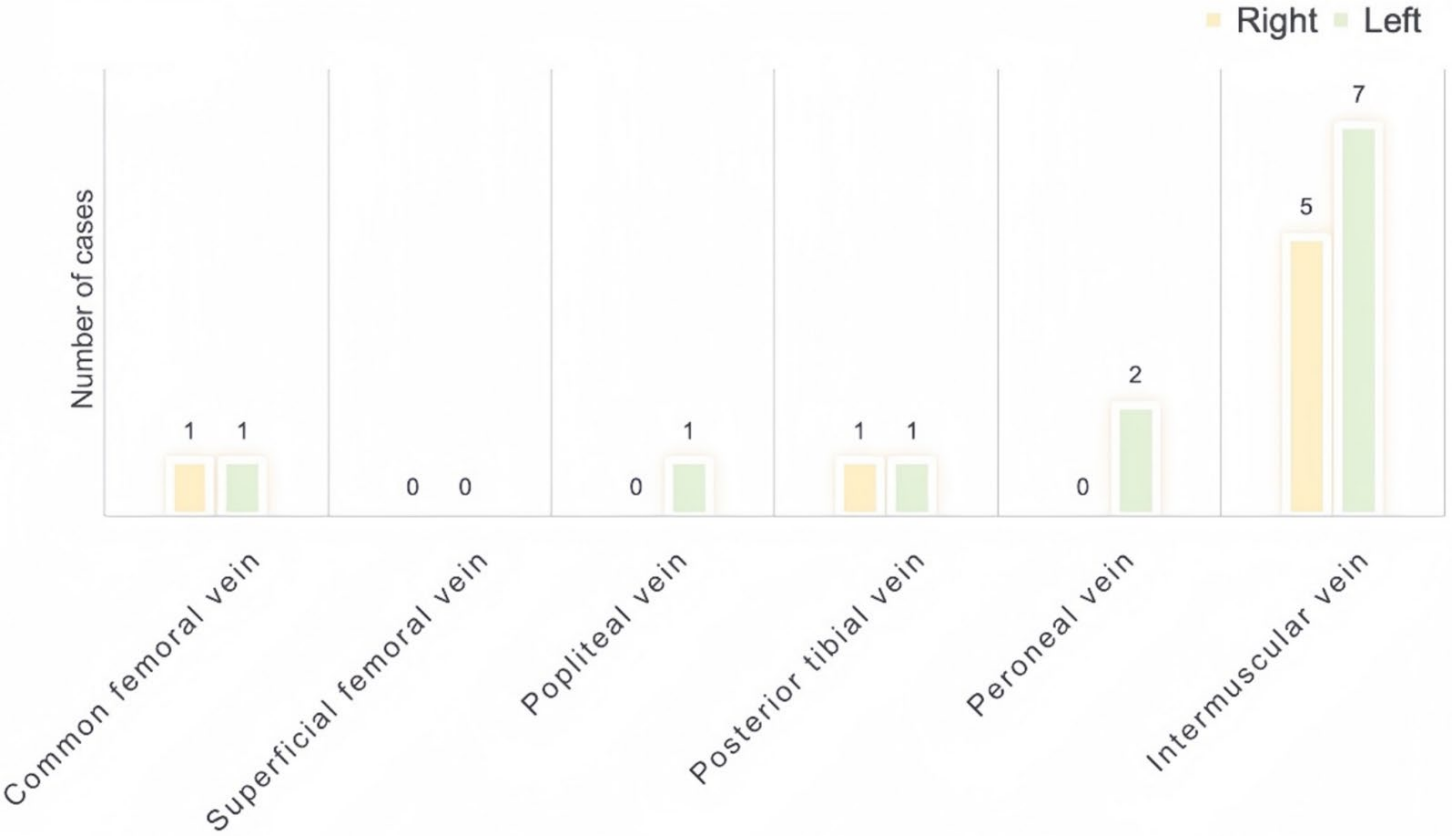
DVT distribution in lower extremity veins. Among the nine confirmed DVT cases, thrombi were most frequently detected in the intermuscular veins, with a predominance on the left side

**Fig. 8:**
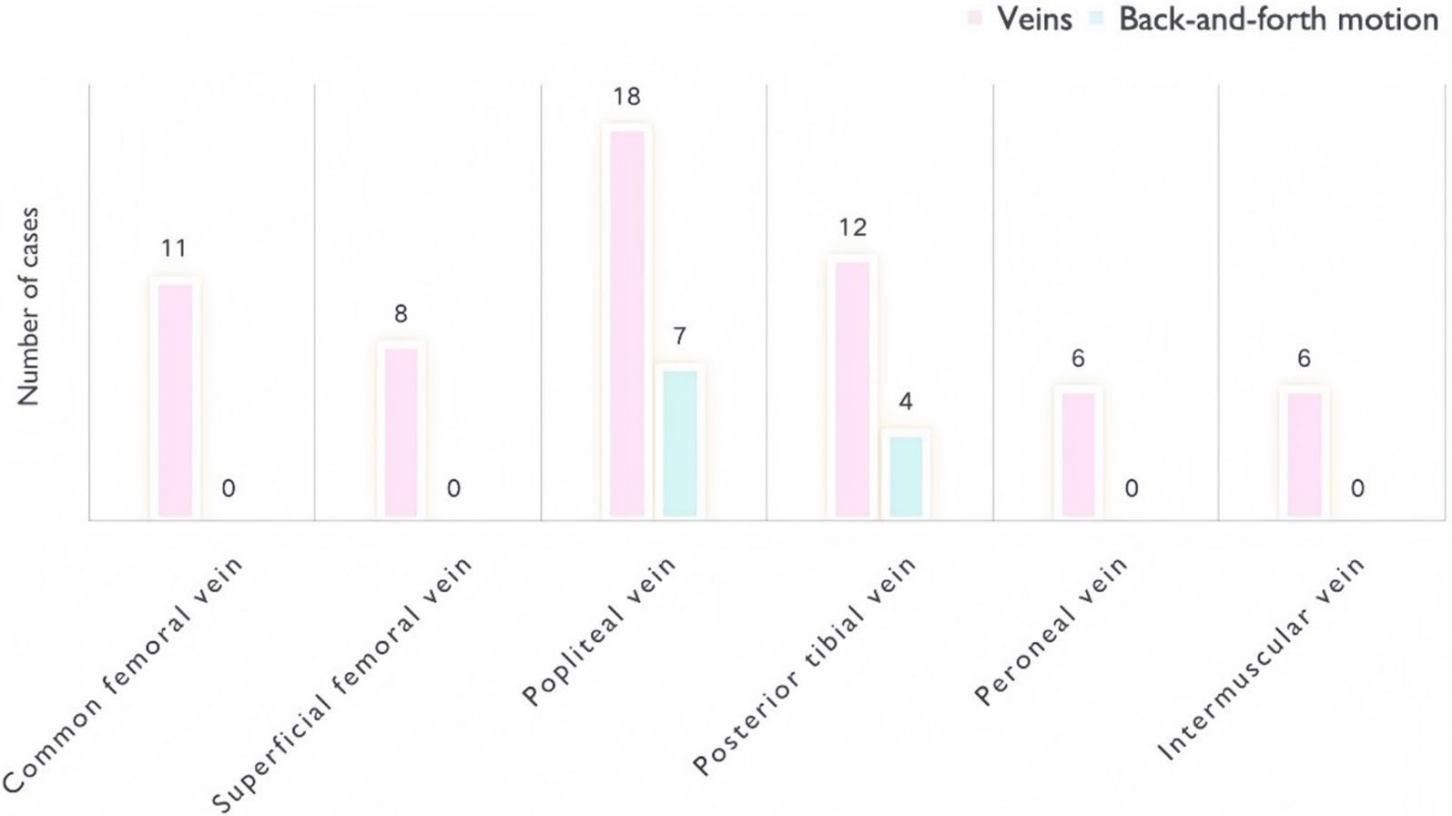
Dynamic characteristics of the “blizzard” sign in DVT patients. Among patients with confirmed DVT, the “blizzard” sign was most frequently observed in the popliteal and posterior tibial veins. Back-and-forth motion was detected in a subset of cases, mainly localized to these two venous segments

DVTs were more frequently detected in the left lower limb, predominantly affecting the intermuscular veins (see Fig. 7). Ultrasound examinations revealed that all cases exhibited a severe, diffuse-type “blizzard” sign, with consistent findings in the popliteal vein across all patients, followed by the posterior tibial vein. The characteristic back-and-forth motion of the “blizzard” sign was also primarily observed in these two veins (see Fig. 8).

## Discussion

This study is among the first to apply nurse-led POCUS for detecting the PTS in postoperative cardiac patients, using the “blizzard” sign as a vascular marker within an interprofessional clinical model. The findings demonstrate a high incidence of PTS (61.8%) in this population, and identify bed rest duration, peak platelet count, and early postoperative CRP level as independent predictors. These results highlight the potential clinical value of incorporating POCUS into routine nursing assessments to facilitate early risk stratification and intervention.

### High incidence and early identification of PTS

Cardiac surgery patients face an elevated risk of thrombotic events due to prolonged operative time, CPB, mechanical ventilation, and postoperative immobility, all of which contribute to venous stasis [2, 10]. Reported incidence of DVT in this population ranges from 0.7% to 48%, reflecting variations in diagnostic methods and patient characteristics [11–13]. This risk is approximately three fold higher than in general surgical procedures [14].

PTS further increases DVT susceptibility. In our study, PTS incidence reached 61.8% among cardiac surgery patients—significantly higher than rates in orthopedic (32.1%) [15] and gynecologic oncology (53.1%) [16] populations. This disparity may stem from unique hemodynamic and inflammatory stressors in cardiac surgery. Notably, the “blizzard” sign, a Doppler ultrasound marker of early venous stasis, was frequently observed in the popliteal and posterior tibial veins, anatomical sites predisposed to thrombus formation due to sluggish flow [17]. Without preventive measures, thrombosis incidence in patients with this sign reaches 66.7% [9]. Thus, the “blizzard” sign serves as a practical, non-invasive indicator for early thrombotic risk, enabling prompt intervention. Given its feasibility, POCUS could enhance routine postoperative assessments, improving PTS detection and management in cardiac surgery patients.

### Clinical predictors of the “blizzard” sign in postoperative cardiac patients

As demonstrated in our results, prolonged bed rest was identified as a primary factor contributing to the development of the “blizzard” sign in postoperative cardiac ICU patients (OR 1.016). Extended immobilization triggered multiple physiological consequences: sedation and mechanical ventilation led to muscle weakness, while gravity-dependent pulmonary secretion retention increased infection risk and delayed ventilator weaning [18]. These effects collectively impaired lower limb mobility, diminished calf muscle pump function, and promoted venous stasis with reduced blood flow velocity, creating a prothrombotic state [19].

Beyond immobility, reactive thrombocytosis within the first 7 postoperative days also emerged as an independent predictor of the “blizzard” sign (OR 1.007). Cardiac surgery induces dynamic platelet fluctuations, typically involving an initial depletion due to CPB, followed by a rebound thrombocytosis within 48 h [20]. This platelet surge, in combination with surgery-induced tissue factor release and coagulation activation [14, 21], substantially elevates thrombotic risk during the early recovery phase.

Consistent with our findings, systemic inflammation, reflected by elevated postoperative CRP levels, was another significant independent predictor of the “blizzard” sign (OR 1.015), underscoring the inflammatory basis of thrombotic risk. CPB, surgical trauma, and ischemia–reperfusion injury all amplify CRP levels, which in turn mediate thrombogenesis through multiple pathways: stimulating inflammatory cytokine production [22], impairing endothelial integrity [23], and enhancing platelet aggregation [24, 25]. Collectively, these effects create a prothrombotic milieu that significantly increases venous thrombosis risk [26].

### Hemodynamic mechanisms and sonographic patterns of PTS

Venous thrombosis frequently originates at valve sites, where turbulent flow and blood stasis promote red blood cell aggregation and local hypercoagulability. In our study, the “blizzard” sign was most commonly observed at the popliteal vein valves—anatomical regions inherently predisposed to early thrombus formation. This echogenic swirling pattern, also known as spontaneous echo contrast (SEC), reflects the accumulation of red blood cell aggregates and plasma proteins near venous valves. Clinically significant SEC, particularly Grade II (nearly complete valve coverage), is considered a pathological PTS and has demonstrated predictive value for subsequent venous thrombosis [27]. Early recognition of SEC via bedside ultrasound may therefore offer a crucial opportunity for timely, preventive intervention before thrombosis becomes clinically apparent.

### Feasibility of nurse-Led POCUS and interprofessional integration

This study underscores the feasibility of nurse-led POCUS for vascular assessment following structured training. Previous studies suggest that with appropriate training, nurses can perform POCUS with a diagnostic accuracy sufficient to support clinical decision-making, especially when integrated within interprofessional care models [28, 29]. Educational interventions further enhance nurses’ confidence and competence in ultrasound use [30], making it realistic to incorporate such skills into clinical nursing roles.

Incorporating nurse-led POCUS into daily ICU assessments may reduce dependence on overburdened imaging departments, shorten diagnostic delays, and enable targeted thromboprophylaxis [31]. A structured protocol—for example, initiating screening within 24–48 h after cardiac surgery for patients at high risk (e.g., prolonged mechanical ventilation, elevated CRP, delayed mobilization)—could support timely identification and doctor review. Such collaborative workflows may improve efficiency, promote individualized prevention strategies, and enhance patient safety.

### Strengths, limitations and implications for nursing practice

To our knowledge, this is the first study to examine the prevalence and predictive value of the “blizzard” sign in postoperative cardiac ICU patients using nurse-led POCUS. The findings suggest that structured ultrasound screening performed by trained nurses can provide a scalable approach for early thrombotic risk detection in this high-risk population, while fostering interprofessional collaboration and nursing-initiated intervention. By enabling bedside identification of early venous stasis, this approach may facilitate personalized thromboprophylaxis and reduce DVT incidence.

This study also has limitations. Patient recovery status and nutritional condition were not systematically assessed. In addition, the single-center design and relatively small sample size may limit generalizability. Future research should focus on multicenter validation, standardization of nurse training programs, and evaluation of clinical outcomes associated with early nurse-led POCUS screening.

## Conclusions

This study identified a high prevalence of PTS among postoperative cardiac ICU patients, with the “blizzard” sign emerging as a feasible and non-invasive sonographic marker for early thrombotic risk. Nurse-led POCUS effectively detected PTS and was successfully implemented through interprofessional collaboration.

Integrating structured nurse-led POCUS protocols into routine nursing workflows may enhance early identification and support individualized thromboprophylaxis. These findings reinforce the expanding role of critical care nurses in advanced clinical assessment and highlight the potential for broader adoption of nurse-initiated vascular screening in intensive care settings. Further research is warranted to validate these findings and inform widespread implementation.

## Supplementary Information

Supplementary Material 1.

Supplementary Material 2.

Supplementary Material 3.

Supplementary Material 4.

Supplementary Material 5.

## Data Availability

The datasets generated and analyzed during the current study are not publicly available due to patient privacy restrictions but are available from the corresponding author on reasonable request.

## Abbreviations

VTE: Venous thromboembolism
PTS: Prethrombotic state
DVT: Deep vein thrombosis
ICU: Intensive care unit
CPB: Cardiopulmonary bypass
POCUS: Point-of-care ultrasound
CCUSG: Chinese Critical Care Ultrasound Study Group
Rcfv: Right common femoral veins
Rsfv: Right superficial femoral vein
Rdfv: Right deep femoral vein
Rpov: Right popliteal vein
BMI: Body mass index
IABP: Intra-aortic balloon pump
CRP: C-reactive protein
SEC: Spontaneous echo contrast
